# Acyclic nucleoside phosphonates containing a second phosphonate group are potent inhibitors of the 6-oxopurine phosphoribosyltransferases and have antimalarial activity

**DOI:** 10.1186/1475-2875-13-S1-P91

**Published:** 2014-09-22

**Authors:** Dianne Keough, Petr Špaček, Dana Hocková, Tomáš Tichý, Silvie Vrbková, Lenka Slavětínská, Zlatko Janeba, Lieve Naesens, Michael Edstein, Marina Chavchich, Tzu Wang, John de Jersey, Luke Guddat

**Affiliations:** 1 School of Chemistry and Molecular Biosciences, University of Queensland, Brisbane, Queensland, Australia; 2 Institute of Organic Chemistry and Biochemistry, Academy of Sciences of the Czech Republic, Prague, Czech Republic; 3 Rega Institue for Medical Research, Katholieke Universiteit Leuven, Leuven, Belgium; 4 Australia Army Malaria Institute, Brisbane, Queensland, Australia

## Background

The 6-oxopurine phosphoribosyltransferases have been suggested to be a target for the discovery of new antimalarial drugs. This is because protozoan parasites rely solely on the salvage of purines from their host to make the nucleotides needed for RNA and DNA synthesis and lack the de novo pathway. Acyclic nucleoside phosphonates (ANPs) that contain a 6-oxopurine base are good inhibitors of the *Plasmodium falciparum* (Pf) and *Plasmodium vivax* (Pv) 6-oxopurine phosphoribosyltransferases (PRTs) [1]. Chemical modifications based on the crystal structure of 2-(phosphonoethoxy) ethylguanine (PEEG) in complex with human HGPRT have led to the design of new ANPs [2]. These novel compounds contain a second phosphonate group attached to the ANP scaffold [3].

## Results

{[(2-[(Guanine-9Hyl)methyl]propane-1,3-diyl)bis(oxy)]bis (me thy-lene)} diphosphonic acid exhibited a K_i_ value of 30 nM for human HGPRT and 70 nM for Pf HGXPRT. The crystal structure of this compound in complex with human HGPRT shows that it fills or partially fills three critical locations in the active site: the binding sites of the purine base, the 5’-phosphate group, and pyrophosphate [3]. This is the first HG(X) PRT inhibitor that has been able to achieve this result. Pro-drugs have been synthesized resulting in IC_50_ values as low as 3.8 μM for Pf grown in cell culture, which is up to 25-fold lower compared to the parent compounds [3].

## Conclusion

The crystal structure of {[(2-[(Guanine-9Hyl)methyl] propane-1,3-diyl)bis(oxy)]bis(methylene)} diphosphonic acid in complex with human HGPRT provides a template for chemical modifications to increase both potency and selectivity for the parasite enzymes.
